# Expert Consensus on the Combined Application of Radiotherapy and Novel Systemic Agents in Breast Cancer Treatment

**DOI:** 10.1111/jebm.70148

**Published:** 2026-06-25

**Authors:** Jie Lan, Wenyi Zhou, Qiufan Zheng, Lu Cao, Wenyan Chen, Zhenyu He, Chunling Jiang, Yanchu Li, Wen Xia, Huihua Xiong, Xiaoli Yu, Jun Zhang, Jianli Zhao, Yanxia Zhao, Lei Liu, Xin Wu, Shusen Wang, Xiaobo Huang, Jing Jing

**Affiliations:** ^1^ Institute of Breast Health Medicine, State Key Laboratory of Biotherapy, West China Hospital, Sichuan University and Collaborative Innovation Center Chengdu China; ^2^ Division of Head and Neck Tumor Multimodality Treatment, Cancer Center, West China Hospital Sichuan University Chengdu China; ^3^ Department of Radiotherapy for Breast Cancer, Yat‐sen Breast Tumor Hospital Sun Yat‐sen Memorial Hospital, Sun Yat‐sen University Guangzhou China; ^4^ Guangdong Provincial Key Laboratory of Malignant Tumor Epigenetics and Gene Regulation Medical Research Center, Sun Yat‐sen Memorial Hospital Guangzhou China; ^5^ Department of Medical Oncology, State Key Laboratory of Oncology in South China, Collaborative Innovation Center for Cancer Medicine Sun Yat‐sen University Cancer Centre Guangzhou China; ^6^ Department of Radiation Oncology Ruijin Hospital Shanghai Jiao Tong University School of Medicine Shanghai China; ^7^ Department of Breast Medicine Nanchang People's Hospital Nanchang China; ^8^ Department of Radiation Oncology, State Key Laboratory of Oncology in South China, Collaborative Innovation Center for Cancer Medicine, Guangdong Key Laboratory of Nasopharyngeal Carcinoma Diagnosis and Therapy Sun Yat‐sen University Cancer Center Guangzhou China; ^9^ Department of Radiation Oncology, Shanghai General Hospital Shanghai Jiao Tong University School of Medicine Shanghai China; ^10^ Department of Oncology, Tongji Hospital, Tongji Medical College Huazhong University of Science and Technology Wuhan China; ^11^ Department of Radiation Oncology Fudan University Shanghai Cancer Center Shanghai China; ^12^ Department of Oncology, Shanghai Medical College Fudan University Shanghai China; ^13^ Shanghai Clinical Research Center for Radiation Oncology Shanghai Key Laboratory of Radiation Oncology Shanghai China; ^14^ Department of Radiation Oncology The Fourth Hospital of Hebei Medical University Shijiazhuang China; ^15^ Breast Tumor Center, Sun Yat‐sen Memorial Hospital Sun Yat‐sen University Guangzhou China; ^16^ Guangdong Provincial Key Laboratory of Malignant Tumor Epigenetics and Gene Regulation, Guangzhou Regenerative Medicine and Health, Guangdong Laboratory, Sun Yat‐sen Memorial Hospital Sun Yat‐sen University Guangzhou China; ^17^ Cancer Center, Union Hospital, Tongji Medical College Huazhong University of Science and Technology Wuhan China; ^18^ Hubei Key Laboratory of Precision Radiation Oncology, Tongji Medical College Huazhong University of Science and Technology Wuhan China

**Keywords:** breast cancer, combination treatment, radiotherapy, systemic agents

## Abstract

The rapid advancement of radiotherapy techniques and systemic anticancer agents has created unprecedented opportunities to improve outcomes for breast cancer patients, while also introducing new challenges related to optimal integration and safety. This consensus, convened by the Breast Cancer Committee of the Chinese Anti‐Cancer Association and Chinese Society of Clinical Oncology Breast Cancer Committee, systematically evaluated available literature through November 2025 and employed Delphi methodology to generate evidence‐based recommendations for combining radiotherapy with immune checkpoint inhibitors, targeted therapies, antibody–drug conjugates and endocrine agents across disease stages. This work aims to guide clinicians toward safer and more effective integration of radiotherapy with contemporary systemic treatments, promote consistency in clinical practice, and identify priority directions for future research to refine precision radiotherapy and systemic therapy combinations in breast cancer.

## 1 Introduction

Breast cancer (BC) is one of the most common malignant tumors among women in China, imposing a substantial health burden, and radiotherapy is a cornerstone of its comprehensive management. In 2022, BC accounted for approximately 357,161 new cases in Chinese women, ranking second among female malignancies in China according to GLOBOCAN data [1]. BC is a biologically heterogeneous disease that can be broadly categorized into hormone receptor (HR)‐positive/human epidermal growth factor receptor 2 (HER2)‐negative disease, HER2‐positive disease, and triple‐negative breast cancer (TNBC), which differ substantially in molecular features, prognosis, and therapeutic vulnerabilities. Recent advances in precision medicine have further refined the treatment landscape of BC, particularly for TNBC, and have highlighted the evolving roles of endocrine therapy (ET), targeted therapy, antibody–drug conjugates (ADCs), and immunotherapy across different subtypes [2].

In parallel, progress in radiotherapy techniques, such as intensity‐modulated radiotherapy (IMRT) and stereotactic body radiotherapy, together with a deeper understanding of biological concepts including immune activation and synergistic radiosensitization with drugs, as well as the rapid development of systemic therapies, have created new opportunities for tumor downstaging, prolonged survival, and improved quality of life. However, clinical challenges persist in the optimized integration of radiotherapy and systemic therapy, including uncertainties in timing and sequencing, absence of definitive combination strategies, inadequate toxicity management, unclear synergistic mechanisms, and limited availability of high‐level evidence [3, 4].

To address these challenges and guide standardized clinical practice in China, Breast Cancer Committee of the Chinese Anti‐Cancer Association and Chinese Society of Clinical Oncology Breast Cancer Committee convened a multidisciplinary consensus panel of experts in breast radiation oncology, surgery, medical oncology, and pharmacy, conducted in‐depth exploration and discussion regarding the series of problems, and developed the Chinese Multidisciplinary Expert Consensus on the Combined Application of Radiotherapy and Novel Systemic Agents in Breast Cancer Treatment.

This consensus outlines fundamental radiotherapy principles while focusing on core issues regarding the combined use of radiotherapy and novel systemic agents. It integrates multiple decision‐making factors, including disease stage, postoperative treatment for early‐stage disease, advanced and palliative settings, and the mechanisms of drug‐radiotherapy synergy. The consensus explores combination strategies across these clinical scenarios, emphasizes the elucidation of synergistic mechanisms, and identifies optimal combination modalities, such as the selection of radiotherapy techniques and drugs, as well as treatment timing and sequencing. Additionally, it establishes a comprehensive framework for toxicity management and proposes directions for future prospective clinical research based on efficacy and safety considerations.

## 2 Methods

### 2.1 Study Registration

In April 2025, a group of experts initiated a collaboration to investigate the attitudes of Chinese professionals toward the combined application of radiotherapy and novel systemic agents in BC treatment. This consensus was registered on December 6, 2025, in the International Practice Guidelines Registry and Transparency Platform and was approved by the platform on December 25, 2025, with the registration number: PREPARE‐2025CN1946. The publicly accessible protocol is available at: https://www.guidelines‐registry.org/my/guidelines/detail/d50f9d02‐874f‐42f1‐ae70‐897989c5cb6b.

### 2.2 Governance Structure and Consensus Panelist Group

A multi‐level governance structure was established to ensure methodological rigor, clinical relevance, and authoritative endorsement. The Steering Committee defined the theme, scope, and core clinical questions; appointed the Chief Advisor Committee, Guiding Expert Committee, and Writing Expert Committee; oversaw the consensus process; selected the Expert Panel; adopted evidence grading and recommendation strength; reviewed the final draft; and approved the consensus for publication. The Chief Advisor Committee organized in‐depth discussions on unresolved recommendations and provided authoritative analysis. The Guiding Expert Committee ensured that all recommendations were clear and actionable. The Writing Expert Committee performed systematic literature searches, reviewed ongoing registered clinical trials, produced the outline, drafted the recommendations, and wrote the consensus. The Expert Panel consisted of specialists in radiation oncology, medical oncology, surgery, and pharmacy, and was responsible for voting on the recommendations to reach consensus.

### 2.3 Systematic Literature Search

A systematic literature search was conducted in PubMed from inception to November 30, 2025, using MeSH terms and free‐text words with Boolean operators. Core terms included “breast neoplasms,” “radiotherapy,” and relevant systemic agents. Ongoing clinical trials and conference abstracts were also reviewed. Original research and systematic reviews on combined radiotherapy and novel systemic agents in breast cancer were included, while comments, animal studies, and duplicates were excluded. Ultimately, 97 articles were included.

### 2.4 Evidence Grading

For each consensus statement, the level of evidence was independently assessed by two methodologists based on the study design of the supporting literature identified in the systematic search. We adopted the GRADE (Grading of Recommendations Assessment, Development and Evaluation) approach to classify the certainty of evidence into four levels: high, moderate, low, and very low. High: We are very confident that the true effect lies close to the estimate of the effect. Moderate: We are moderately confident in the effect estimate; the true effect is likely to be close to the estimate, but there is a possibility that it is substantially different. Low: Our confidence in the effect estimate is limited; the true effect may differ substantially from the estimate. Very low: We have very little confidence in the effect estimate; the true effect is likely to be substantially different from the estimate. For statements where no direct published evidence was available from the systematic search, the certainty of evidence was classified as very low.

### 2.5 Consensus Process

The board collaboratively developed a set of questions and statements on therapeutic strategies for combining radiotherapy with novel systemic agents. A two‐round Delphi process was used [5, 6]. In the first round, statements with ≥80% agreement were revised and advanced to the second round; those with <80% agreement were revised and voted again. In the second round, statements achieving ≥80% agreement were accepted as final consensus; others were discarded. Recommendation strength was based on GRADE: strong for high/moderate certainty, conditional for low/very low certainty. The final version was approved by the scientific board. The survey was conducted via an online platform; consent was implied by completion with anonymous voting. Participation was voluntary.

## 3 Results

A total of 28 consensus statements were developed through a two‐round Delphi process. These statements cover the combined use of radiotherapy with ET, targeted therapy (including anti‐HER2 agents, CDK4/6 inhibitors, PARP inhibitors, PI3K/AKT/mTOR pathway inhibitors), ADCs (T‐DM1, T‐DXd, Sacituzumab govitecan (SG) and novel TROP‐2 ADCs), ICIs (Pembrolizumab, Toripalimab, Camrelizumab), anti‐angiogenic agents (Bevacizumab, Apatinib), and selective estrogen receptor degraders (SERDs). The recommendations address both early‐stage and advanced/palliative settings, with specific considerations for intracranial versus extracranial lesions.

**FIGURE 1:**
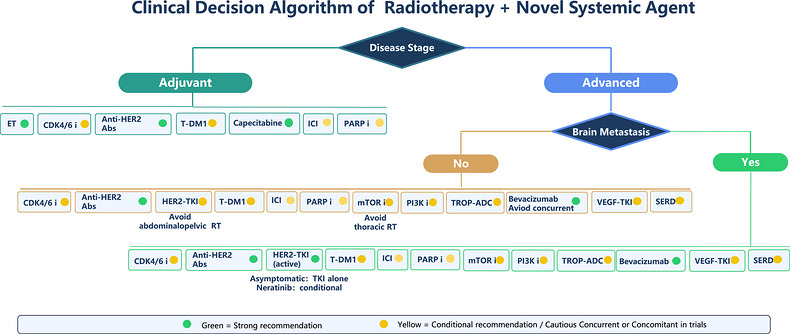
Clinical decision algorithm of radiotherapy + novel systemic agent.

To facilitate clinical decision‐making, we developed a decision algorithm (Figure 1) that guides the choice among concurrent, concomitant, sequential, or deferred radiotherapy based on drug class. Table 1 outlines the timing recommendations: concurrent is defined as radiotherapy and systemic agents administered with a time interval of <5 drug elimination half‐lives; concomitant as radiotherapy and systemic agents given within the same overall treatment course but with an interval of ≥5 drug elimination half‐lives. Table 2 presents a framework for toxicity monitoring. The following sections present each statement with supporting evidence and practical considerations.

**TABLE 1:** timing recommendations for combined radiotherapy and systemic agents.^*^

Drug class	Specific agent	Disease stage	Irradiation site/condition	Timing recommendation	Consensus No.	Strength of recommendation	Remarks/evidence level
Endocrine therapy (ET)	Tamoxifen/AI	Adjuvant	Postoperative RT	Concomitant or sequential	1	Strong	Well‑tolerated; no need to interrupt ET
	Tamoxifen/AI	Adjuvant, age ≥65 years, low‐risk, limited life expectancy	Postoperative RT	Omission postoperative RT	2	Strong	Adjuvant ET is necessary
	Tamoxifen/AI			Postoperative RT alone	2	Strong	Intolerant to ET
CDK4/6 inhibitors	Abemaciclib/Ribociclib/Dalpiciclib	Adjuvant	Postoperative RT	Sequential preferred; concurrent allowed only in clinical trials	3	Conditional	No high‑level evidence for concurrent use
	Abemaciclib/Ribociclib/Dalpiciclib	Advanced	Oligometastatic lesions	Concomitant	19	Conditional	Manageable toxicity; effective pain relief
	Abemaciclib/Ribociclib/Dalpiciclib	Advanced	Pelvis/multiple vertebrae	Cautious concomitant; monitor hematologic toxicity	19	Conditional	Increased risk of hematologic toxicity
	Abemaciclib/Ribociclib/Dalpiciclib	Advanced	Intestinal irradiation (abdominal/pelvic)	Withhold CDK4/6i (especially Abemaciclib)	19	Conditional	Risk of severe diarrhea
Anti‑HER2 antibodies	Trastuzumab ± Pertuzumab	Adjuvant	Postoperative RT	Concomitant	4	Strong	NCCTG N9831/APHINITY support
	Trastuzumab ± Pertuzumab	Brain metastases	WBRT/SRT/SRS	Concomitant	10	Strong	No significant increase in radionecrosis
	Trastuzumab ± Pertuzumab	Extracranial	Bone/oligometastases	Concomitant	10	Strong	Monitor cardiac function
	Inetetamab	Adjuvant	Postoperative/RT/WBRT/SRT/SRS	Cautious concomitant	11	Conditional	Similar to Trastuzumab, but lack of evidence
	Margetuximab						
T‑DM1	Trastuzumab emtansine	Adjuvant	Postoperative RT	Concomitant	5	Conditional	Monitor platelets
	Trastuzumab emtansine	Brain metastases	SRS/SRT	Avoid concomitant	16	Conditional	Increased risk of radionecrosis
	Trastuzumab deruxtecan	Brain metastases	SRS	Avoid concomitant	17	Conditional	Increased risk of radionecrosis
	Trastuzumab deruxtecan	Advanced extracranial	Oligometastases	Concomitant	18	Conditional	Avoid thoracic radiotherapy
HER2‑TKI	Pyrotinib	Asymptomatic brain metastases	Low tumor burden	Avoid concomitant	12	Strong	Especially for not previously received HER2 TKI patient
	Pyrotinib	Active brain metastases	SRT/SRS	Concomitant	12	Strong	Improves intracranial control
	Tucatinib	Advanced brain metastases	SRT	Concurrent	13	Strong	HER2CLIMB trial support
	Neratinib	Brain metastases	SRT/SRS	Cautious concomitant in trials	14	Conditional	Lack of evidence
	Pyrotinib/Tucatinib/Neratinib	Advanced	Bone/oligometastases	Cautious concomitant	15	Conditional	Similar to brain metastases, but lack of evidence
			Abdominopelvic	Avoid concomitant	15	Conditional	Diarrhea should be emphasized
PARP inhibitor	Olaparib	Adjuvant	Postoperative RT	Sequential recommended	9	Conditional	OlympiA design; RADIOPARP showed toxicity
	Olaparib	Advanced	Any site	Sequential; cautious concomitant only with small‑field low‑dose RT in trials	20	Conditional	Insufficient evidence for routine concurrent use
Capecitabine	Capecitabine	Adjuvant	Postoperative RT	Concurrent	6	Strong	Improves survival; manageable toxicity
Immune checkpoint inhibitor (ICI)	Pembrolizumab	Neoadjuvant	Breast tumor bed	Cautious concurrent in trials	8	Conditional	Research setting preferred, NCT06769919, NCT06627712 are on going
	Pembrolizumab	Adjuvant	Postoperative RT	Concurrent (hypofractionation preferred)	7	Conditional	KEYNOTE‑522 safety analysis, NCT07046195 is on going
	Pembrolizumab/Toripalimab/Camrelizumab	Advanced	Any site	Cautious concurrent in trials	23	Conditional	Research setting preferred, NCT06735131 is on going
mTOR inhibitor	Everolimus	Advanced	Thoracic RT	Not recommended	21	Conditional	Increased risk of pulmonary toxicity
PI3K inhibitor	Alpelisib/Inavolisib	Advanced	Any site	Cautious concomitant in trials	22	Conditional	Research setting preferred
AKT inhibitor	Capivasertib	Advanced	Any site	Cautious concomitant in trials	22	Conditional	Research setting preferred
TROP‑2 ADC	Sacituzumab govitecan	Advanced	Bone/brain	Concurrent (clinical trials are preferred)	24	Conditional	Retrospective data support, for brain: enhanced RN monitoring
	SKB‐264/Dato‐DXd	Advanced	Bone/brain	Cautious concomitant in trials	25	Conditional	Lack of evidence
Anti‑VEGF	Bevacizumab	Brain metastases	WBRT/SRT	Concomitant	26	Strong	Reduces edema; improves PFS
	Bevacizumab	Advanced extracranial	Lung/gastrointestinal RT	Not recommended	26	Strong	Risk of perforation/pneumonitis
	Apatinib	Brain metastases	Brain	Cautious concomitant in trials	27	Conditional	Insufficient evidence
SERD	Elacestrant	Advanced	Bone/oligometastases	Cautious concomitant in trials	28	Conditional	No overlapping toxicity Insufficient evidence

Concurrent: Radiotherapy and systemic agents are administered with a time interval of <5 drug elimination half‐lives. Concomitant: Radiotherapy and systemic agents are given within the same overall treatment course, but the time interval is ≥5 drug elimination half‐lives.

* Novel systemic agents in this article refer to Capecitabine, ET, ADCs, CDK4/6 inhibitors, PARP inhibitors, ICIs, and other agents recently integrated with radiotherapy.

**TABLE 2:** Toxicity monitoring for combined radiotherapy and systemic agents.

Drug + RT combination	Main toxicity	Monitoring parameters	Monitoring frequency	Intervention threshold	Management recommendations
CDK4/6i + chest wall/whole breast RT	Radiation pneumonitis, cytopenias	Symptoms (cough, dyspnea), CBC(complete blood count), chest CT	Every week during RT, then 3 months post‑RT	Grade ≥2 pneumonitis or Grade ≥3 cytopenias	Withhold CDK4/6i; corticosteroids
CDK4/6i + pelvic/multiple vertebral RT	Cytopenias	CBC	Weekly	Grade 3 cytopenias	Withhold CDK4/6i; G‐CSF or rhTPO if needed
CDK4/6i + abdominal/intestinal RT	Diarrhea	Stool frequency, electrolytes	Daily assessment	Grade ≥2 diarrhea	Withhold CDK4/6i (especially Abemaciclib); hydration, Loperamide
T‐DM1 + postoperative RT	Cytopenias, radiation dermatitis	CBC, skin reaction	Weekly	Platelets <50×10^9^/L or Grade ≥3 dermatitis	Hold T‐DM1; dose reduction or delay
T‐DM1 + SRS	Radiation necrosis	Neurologic symptoms, MRI	Every 3 months for 1 year	New edema or enhancement	Hold T‐DM1
T‐DXd + SRS	RN, interstitial lung disease	Neurosymptoms, respiratory symptoms	Every 3 months for MRI, every 6–12 weeks for CT	Any grade ILD or symptomatic RN	Hold T‐DXd; corticosteroids; permanent discontinuation for Interstitial lung disease
Pyrotinib + SRT	Diarrhea, radiation necrosis	Stool frequency, neurosymptoms	Diarrhea: daily; every 3 months (brain MRI)	Grade ≥2 diarrhea or RN	Antidiarrheals, dose reduction; for radiation necrosis, hold TKI
PARP inhibitor + RT	Dermatitis, cytopenias	Skin examination, CBC	Weekly	Grade ≥3 dermatitis or cytopenias	Hold PARP inhibitor; reduce dose (e.g., Olaparib 25 mg BID)
Capecitabine + postoperative RT	Hand‐foot syndrome, radiation dermatitis	Skin (palms, soles, irradiated field)	Every week	Grade ≥2 hand‐foot or dermatitis	Hold Capecitabine; topical care
Pembrolizumab + RT	Dermatitis, pneumonitis, thyroid dysfunction	Skin, respiratory symptoms, thyroid function	Every 3 weeks (prior to ICI)	Grade ≥3 immune‐related AE	Hold ICI; corticosteroids
Bevacizumab + WBRT/SRT	Hypertension, proteinuria, bleeding	Blood pressure, urinalysis, bleeding signs	Weekly (BP), monthly (urine)	BP >160/100 or proteinuria ≥2+	Antihypertensives; hold Bevacizumab
Sacituzumab govitecan + RT	Cytopenias, diarrhea, radiation necrosis (if brain)	CBC, stool frequency, brain MRI if indicated	Weekly (CBC), every 3 months (brain MRI)	Grade 3 cytopenias or radiation necrosis	G‐CSF; hold SG; for RN, standard management

## 4 Consensus

### 4.1 Overall Principle for Postoperative Radiotherapy

Postoperative radiotherapy for breast cancer primarily targets the whole breast ± tumor bed/chest wall and/or regional lymph node drainage areas. The main organs at risk include the skin within the radiation field, lungs, heart, liver, thyroid, esophagus, and others. The key to combining radiotherapy with systemic therapy lies in optimizing the timing and sequencing. While ensuring adequate toxicity management and safety and avoiding overlapping toxicities between radiotherapy and systemic agents, further exploration is needed to identify optimal combination regimens and synergistic efficacy.

#### 4.1.1 Adjuvant Endocrine Therapy (ET)

Adjuvant ET reduces the risk of recurrence and improves overall survival (OS) for BC patients. For patients with early‐stage invasive ductal carcinoma, guidelines explicitly state that ET can be administered concomitantly with radiotherapy [7]. Currently, no studies have specifically explored the timing of radiotherapy and ET for ductal carcinoma in situ (DCIS) patients after breast‐conserving surgery (BCS); therefore, clinical practice generally refers to the safety data established for early‐stage invasive BC.

The omission of radiotherapy in elderly patients with low‐risk HR‐positive BC after BCS remains a subject of debate. The CALGB‐9343 and PRIME II trials demonstrated that while radiotherapy does not improve OS, it significantly reduces local recurrence [8, 9]. Consequently, individualized decision‐making should integrate recurrence risk, comorbidities, life expectancy, patient preference, and the potential impact on quality of life (QoL). For patients with a long life expectancy and good functional status, radiotherapy combined with ET is recommended. For those with significant comorbidities or limited life expectancy, omission of radiotherapy may be considered, acknowledging that ET alone may be associated with declines in QoL (e.g., vasomotor symptoms, arthralgias, fatigue) and that this trade‐off should be discussed with the patient. For patients with poor tolerance to ET, accelerated partial breast irradiation (APBI) or ultra‐hypofractionated radiotherapy (referencing the EUROPA study) may serve as an alternative to ET [10].

**Consensus 1: Based on its favorable safety and tolerability profile, this consensus recommends that radiotherapy and adjuvant ET be administered either concomitantly or sequentially following BCS for ER+ early invasive BC or DCIS. For ER+ patients who have already initiated ET before radiotherapy with good tolerance, there is no need to interrupt the continuity of ET during the radiotherapy period**.

**Consensus 2: For elderly patients (age ≥65 years) with low‐risk HR‐positive/HER2‐negative pT1‐2N0M0 BC, individualized treatment decisions should be guided by a comprehensive assessment of recurrence risk, comorbidities, life expectancy, tolerance to radiotherapy and ET, patient preference, and QoL considerations. This consensus suggests that omission of postoperative radiotherapy may be considered in patients with significant comorbidities and limited life expectancy, provided adjuvant ET is continued and after a shared decision‑making process that explains the potential QoL trade‑offs of ET alone. Conversely, radiotherapy alone may be considered for those intolerant to ET. When radiotherapy is indicated, ultra‐short‐course regimens (such as APBI or whole‐breast ultra‐hypofractionated radiotherapy) are preferred due to their lower toxicity and better quality of life**.

#### 4.1.2 Adjuvant CDK4/6 Inhibitors With ET

Cyclin‐dependent kinase 4 and 6 (CDK4/6) inhibitors combined with ET have emerged as a critical adjuvant intensification strategy for early‐stage HR‐positive BC patients with intermediate‐to‐high risk. Numerous randomized clinical trials demonstrated that CDK4/6 inhibitors significantly improve invasive disease‐free survival (DFS) in this population [11, 12, 13]. However, in these trials, CDK4/6 inhibitors were administered sequentially after adjuvant radiotherapy. A follow‐up analysis of the MonarchE study showed that the incidence of radiation pneumonitis (RP) was similar between the Abemaciclib and placebo groups among patients who had previously received radiotherapy [14]. Emerging evidence suggests that moderately hypofractionated regimens (e.g., 40 Gy/15 fx for breast/chest wall) are associated with lower rates of acute and late toxicities, particularly RP and esophagitis. Such low‐toxicity fractionated schedules, when combined with CDK4/6 inhibitors, may theoretically reduce the risk of overlapping toxicities and thus enable safe concurrent administration. Several ongoing prospective studies (e.g., NCT05656794, NCT05813756) are specifically evaluating concurrent adjuvant radiotherapy using hypofractionated intensity‐modulated techniques with Abemaciclib or Ribociclib.

**Consensus 3: Based on lack of high‐level evidence regarding the combined application of postoperative radiotherapy and CDK4/6 inhibitors, this consensus recommends sequential treatment with radiotherapy followed by CDK4/6 inhibitors. However, the favorable safety profile of concurrent CDK4/6 inhibitors with radiotherapy in advanced disease, the availability of low‐toxicity moderate hypofractionated regimens, and ongoing prospective adjuvant trials suggest that concurrent administration may be considered under specific circumstances—ideally within prospective clinical trials or under strict multidisciplinary management. When concurrent therapy is pursued, moderately hypofractionated radiotherapy is preferred to minimize toxicity, and strict monitoring for hematological toxicity and other relevant adverse events (AEs) is essential, especially during the first two weeks of CDK4/6 inhibitor therapy. Prospective clinical studies are encouraged to validate the safety and potential synergy of concurrent low‐toxicity fractionated radiotherapy with adjuvant CDK4/6 inhibitors**.

#### 4.1.3 Adjuvant Anti‐HER2 Therapy (Trastuzumab/Pertuzumab)

Anti‐HER2 therapy has markedly improved the prognosis of HER2‐positive BC. In clinical practice, concomitant administration of postoperative radiotherapy and Trastuzumab is common, though potential cardiotoxicity requires attention. The NCCTG N9831 study showed that concomitant radiotherapy did not increase Grade≥3 cutaneous or cardiac AEs [15]; long‐term follow‐up from the HERA trial also observed no significant decline in left ventricular function or increased cardiovascular events [16]. The APHINITY trial confirmed that dual HER2 blockade (Trastuzumab and Pertuzumab) further improves outcomes, with no additional safety signals observed during concurrent radiotherapy in clinical settings.

**Consensus 4: Based on key clinical studies that have demonstrated favorable safety and tolerability, this consensus recommends concomitant application of postoperative radiotherapy with Trastuzumab/Pertuzumab dual‐targeted adjuvant therapy. For elderly patients with left‐sided BC or those with cardiovascular disease or cardiac dysfunction, it is essential to assess and control cardiovascular risk factors, with targeted therapy recommended sequentially in such cases. During radiotherapy, hypofractionated IMRT, respiratory gating, or proton therapy should be employed to minimize cardiac dose, alongside close monitoring of cardiac function**.

#### 4.1.4 Adjuvant T‐DM1 for HER2‐Positive Non‐pCR

For HER2‐positive patients who do not achieve a pathological complete response (pCR) after neoadjuvant therapy, the KATHERINE trial established that adjuvant intensification with Trastuzumab emtansine (T‐DM1) significantly reduces recurrence risk and is permissible concurrently with radiotherapy. Safety analyses showed that the incidence of radiation dermatitis and Grade ≥3 dermatitis was comparable to Trastuzumab; while RP increased slightly, the overall incidence remained low [17]. The ATEMPT study also suggested that concomitant treatment might increase skin toxicity, though the difference was not statistically significant, and radiotherapy fractionation may influence toxicity profiles [16].

**Consensus 5: Based on key clinical studies that have shown no significant overlapping toxicity between postoperative radiotherapy and T‐DM1, this consensus suggests that postoperative radiotherapy and T‐DM1 may be administered concomitantly. Hypofractionated radiotherapy regimens are recommended to reduce acute skin toxicity. Platelet levels should be closely monitored during treatment, and if severe AEs such as Grade 3–4 thrombocytopenia occur, concomitantly use should be avoided thereafter. Furthermore, attention should be paid to emerging evidence on the advantage of Trastuzumab deruxtecan (T‐DXd) in adjuvant intensification therapy, and prospective studies on the safety and efficacy of combining postoperative radiotherapy with next‐generation ADCs are encouraged**.

#### 4.1.5 Adjuvant Capecitabine for TNBC

TNBC accounts for approximately 15%‐20% of invasive BCs. For TNBC patients with residual disease after neoadjuvant therapy, adjuvant Capecitabine is standard, given its demonstrated survival benefit [18]. Additionally, some high‐risk patients may receive metronomic Capecitabine for further intensification following standard adjuvant chemotherapy [19]. Regarding the timing of radiotherapy and Capecitabine, emerging clinical data support the feasibility of concurrent administration. A retrospective study reported at ASCO 2024 evaluated concurrent Capecitabine with radiotherapy in patients with locally advanced TNBC and residual disease after neoadjuvant therapy. The regimen yielded favorable survival outcomes, although a higher incidence of Grade ≥3 radiation dermatitis underscores the need for close toxicity monitoring and management [20]. A Phase II single‐arm prospective study also showed that concurrent radiotherapy and Capecitabine was tolerable in TNBC patients with residual disease [21]. Further supporting this approach, a Korean multicenter retrospective study demonstrated that concurrent regimen was associated with improved DFS, OS, and distant metastasis control compared to sequential therapy, without increasing Grade ≥2 toxicities or treatment discontinuation rates [22].

**Consensus 6: Based on the superior survival outcomes (improved DFS, OS, and distant metastasis control) of concurrent over sequential therapy, along with its acceptable safety, concurrent radiotherapy and Capecitabine are recommended for TNBC patients who have residual disease after neoadjuvant therapy or who receive metronomic Capecitabine after standard adjuvant chemotherapy**.

#### 4.1.6 Adjuvant Immunotherapy (Pembrolizumab) for TNBC

The KEYNOTE‐522 study established a treatment paradigm incorporating neoadjuvant Pembrolizumab plus chemotherapy followed by adjuvant Pembrolizumab, significantly improving pCR and event‐free survival [23]. In patients requiring postoperative radiotherapy, the protocol permitted both concurrent and sequential administration of adjuvant Pembrolizumab. Safety analyses demonstrated low rates of severe treatment‐related AE regardless of timing, supporting the tolerability of this combination in the radiotherapy setting [24]. Real‐world evidence also supports this conclusion. A retrospective study from Institute Curie compared radiotherapy plus Pembrolizumab versus radiotherapy alone and found similar rates of radiation dermatitis, fatigue, and lymphoedema, suggesting acceptable safety for adjuvant radio‐immunotherapy [25]. However, the definitive benefit, optimal timing, and target population for adding ICIs to radiotherapy in the postoperative setting remain limited, with ongoing randomized trials expected to clarify these questions (NCT07046195).

**Consensus 7: Based on its favorable safety and tolerability profile, this consensus recommends that concurrent administration of radiotherapy and Pembrolizumab may be considered in the adjuvant setting, with hypofractionated radiotherapy regimens preferred for their enhanced immune‐activating effects. For other programmed cell death protein 1 (PD‐1) or programmed death‐ligand 1 (PD‐L1) monoclonal antibodies, this consensus recommends prospective clinical studies to further elucidate optimal sequencing, identify beneficiary populations, and validate the safety of such combinations**.

#### 4.1.7 Neoadjuvant Radio‐Immunotherapy for TNBC

Building on the KEYNOTE‐522 framework, integrating neoadjuvant radiotherapy with immunotherapy represents a promising avenue to improving TNBC outcomes. Early‐phase studies suggest that short‐course radiotherapy combined with ICIs and chemotherapy yields high pCR rates with Grade≥3 AEs remain primarily driven by chemotherapy‐related toxicity [26]. Multiple Phase III randomized trials are currently ongoing to further validate the efficacy and safety of this approach (NCT06769919, NCT06627712).

**Consensus 8: Although high‐level evidence of neoadjuvant PD‐1/PD‐L1 inhibitors with radiotherapy is currently lacking, this approach represents a promising treatment strategy and an important research direction. This consensus encourages conducting clinical studies on preoperative radio‐immunotherapy to elucidate its efficacy and to identify beneficiary populations and relevant clinical biomarker parameters**.

#### 4.1.8 Adjuvant PARP Inhibitors (Olaparib) for gBRCA‐Mutated TNBC

Based on the OlympiA trial, Olaparib is used for high‐risk TNBC patients with germline breast cancer susceptibility gene (gBRCA) mutations who previously received neoadjuvant or adjuvant chemotherapy [27]. However, the study required radiotherapy to be completed prior to enrollment and provided no evidence for the concomitant use of adjuvant radiotherapy and PARP inhibitors. In terms of exploratory research, the RADIOPARP Phase I dose‐escalation trial evaluated the safety of concurrent Olaparib and adjuvant radiotherapy in early‐stage high‐risk TNBC. 50.4 Gy/28 fx to the whole breast, 50 Gy/25 fx to the chest wall, and 50–50.4 Gy to regional nodes. In this single‐arm trial, Olaparib was dose‐escalated (50 mg, 100 mg, 150 mg, and 200 mg twice daily), with a total of 24 patients treated. Among the reported Grade 3–4 acute non‐dose‐limiting toxicities, Grade 3 radiation dermatitis occurred in 2 patients (8.3%) and Grade 3 lymphopenia in 8 patients (33.3%), while Grade 4 lymphopenia was observed in 3 patients (12.5%) [28]. With a median follow‐up of 34 months, the 3‐year event‐free survival was 65% and OS 83% [28]. Given the limited sample size, these results should be interpreted cautiously, and large‐scale prospective trials are needed to further evaluate the safety and efficacy of concurrent adjuvant radiotherapy and PARP inhibitors.

**Consensus 9: Based on the insufficient evidence on the efficacy and safety of combining radiotherapy with PARP inhibitors in the adjuvant setting, this consensus recommends sequential administration of radiotherapy followed by PARP inhibitors during adjuvant therapy. For high‐risk TNBC patients (e.g., non‐pCR, pT2 or higher, or pN+) with gBRCA mutations, exploration of optimal combined modalities of radiotherapy and PARP inhibitors in the adjuvant setting may be considered within the framework of clinical trials**.

### 4.2 Overall Principle for Advanced BC

In the management of advanced breast cancer, molecular subtype‐guided systemic therapy remains the cornerstone of treatment. Radiotherapy may be thoughtfully incorporated into the systemic treatment framework to address local disease, with the goal of alleviating symptoms, offering added therapeutic benefit, and in selected cases, contributing to a potential cure. When considering radiotherapy, efforts should be made to avoid unnecessary disruption of systemic therapy, respect patient tolerance, and ensure rigorous control of treatment‐related toxicities. The following consensus statements offer practical guidance on the concurrent or sequential use of radiotherapy with various systemic agents in the advanced setting, with a focus on individualized, patient‐centered decision‐making.

#### 4.2.1 Advanced HER2‐Positive: Trastuzumab and Pertuzumab

Trastuzumab with or without Pertuzumab combined with Taxanes is a standard first‐line regimen for advanced HER2‐positive BC, and its use with radiotherapy is common. While basic research suggests radiosensitization [29, 30], clinical decisions should be guided by safety and local control data. Radionuclide‐labeling studies suggest that these agents can cross the blood‐brain barrier to some extent, providing supportive evidence for combination with radiotherapy for brain metastases [31, 32]. For intracranial lesions, retrospective data suggest that dual HER2 blockade is tolerable when used concurrently with whole‐brain radiotherapy (WBRT), stereotactic radiotherapy (SRT), or stereotactic radiosurgery (SRS). Small‐sample data indicate that fractionated SRT concurrent with dual blockade achieves a high objective response rate (ORR) with low rates of radiation necrosis (RN) [33]. A larger retrospective analysis of SRS showed that concurrent anti‐HER2 therapy, particularly with Pertuzumab, was associated with improved local control and OS signals without a significant increase in symptomatic radiation‐related AEs, although the overall rate of radiation‐related AEs might increase [34]. For extracranial lesions, several retrospective studies suggest that the safety of concurrent palliative or radical radiotherapy with Trastuzumab/Pertuzumab is manageable; cardiotoxicity usually presents as mild, asymptomatic left ventricular ejection fraction (LVEF) reduction, necessitating routine monitoring and adherence to cardiac dose constraints [35, 36, 37].

**Consensus 10: Based on its favorable safety and tolerability profile, this consensus recommends that Trastuzumab and/or Pertuzumab may be administered concomitantly with WBRT or intracranial SRT, as their combination does not increase safety risks, and evidence suggests potential benefits of combining Pertuzumab in patients with brain metastases. For extracranial lesions, such as oligometastatic disease requiring palliative radiotherapy for bone metastases, concomitant radiotherapy may be considered in addition to systemic therapy based on clinical needs**.

#### 4.2.2 Advanced HER2‐Positive: Inetetamab and Margetuximab

Inetetamab and Margetuximab are Fc‐engineered anti‐HER2 monoclonal antibodies, but clinical evidence for their use with radiotherapy remains limited, lacking prospective randomized trials or large‐scale real‐world data. Given their comparable framework and toxicity management pathways to Trastuzumab, clinical strategies may refer to Trastuzumab‐related evidence and monitoring protocols.

**Consensus 11: Given that Inetetamab and Margetuximab are highly similar to Trastuzumab in terms of target binding, signal inhibition, and activation mechanisms, this consensus recommends that the clinical recommendations and safety management for their combination with radiotherapy should refer to those established for Trastuzumab**.

#### 4.2.3 Advanced HER2‐Positive: Pyrotinib for Brain Metastases

Pyrotinib is an oral irreversible HER2 tyrosine kinase inhibitors (TKIs) widely used in HER2‐positive advanced BC, primarily integrated with radiotherapy for brain metastases. While basic research suggests radiosensitization, the key clinical questions are: which patients can delay radiotherapy with systemic therapy alone, and which require concurrent radiotherapy to improve local control [38]. For asymptomatic patients with low intracranial tumor burden, the PERMEATE study showed that Pyrotinib plus Capecitabine yields high intracranial response rates and manageable toxicity, supporting frontline systemic therapy to delay or avoid cranial radiotherapy in selected patients. If radiotherapy is required, the BROPTIMA Phase II single‐arm study showed that Pyrotinib (with or without Capecitabine) combined with SRT or WBRT is safe and achieves good intracranial control [39]. Multiple retrospective studies also suggest that combined therapy improves response and survival without significant new neurotoxicity signals [40, 41, 42, 43, 44]. Some data suggest concurrent administration may be superior to sequential, particularly for patients with active brain metastases [44, 45]. Systematic evidence for concurrent extracranial radiotherapy and Pyrotinib is currently lacking.

**Consensus 12: For HER2‐positive BC patients with brain metastases who are asymptomatic, have a low tumor burden, and have not previously received HER2‐TKIs therapy, this consensus suggests prioritizing systemic therapy based on Pyrotinib and considering postponing or avoiding brain radiotherapy. For patients requiring brain radiotherapy, particularly those with active brain metastases, concomitant use of Pyrotinib is recommended, as this combination has demonstrated favorable efficacy and safety, with evidence suggesting potential survival benefits. SRT is the preferred radiation technique**.

#### 4.2.4 Advanced HER2‐Positive: Tucatinib for Brain Metastases

Tucatinib is a highly selective HER2‐TKIs. It significantly improved outcomes for patients with brain metastases in the HER2CLIMB study, establishing its critical role in HER2‐positive brain metastasis [46]. Retrospective studies suggest that Tucatinib combined with SRS achieves high local control rates with acceptable RN incidence, providing a clinical basis for integration with cranial radiotherapy [47].

**Consensus 13: Based on its favorable safety and tolerability profile, this consensus recommends that Tucatinib be combined with brain radiotherapy in the treatment of brain metastases, with SRT being the preferred radiation technique. Concurrently, prospective clinical studies on such combination regimens are recommended**.

#### 4.2.5 Advanced HER2‐Positive: Neratinib for Brain Metastases

Neratinib has clear intracranial activity in patients with brain metastases, but there is currently a lack of prospective or systematic retrospective evidence evaluating its concurrent or sequential use with WBRT or SRT. Clinical use with radiotherapy should acknowledge this evidence gap and prioritize exploration within research frameworks.

**Consensus 14: Based on the lack of clinical evidence for combining of Neratinib with brain radiotherapy, but due to its mechanistic similarity to other HER2‐TKIs such as Pyrotinib and Tucatinib, as well as the demonstrated tolerability and potential efficacy of the latter two in combination with radiotherapy. This consensus suggests that the combination of Neratinib with brain radiotherapy should be used cautiously and primarily explored within clinical trials**.

#### 4.2.6 Advanced HER2‐TKIs: Extracranial Palliative Radiotherapy

Regarding palliative or radical radiotherapy for extracranial lesions such as oligometastases, evidence for combination with Pyrotinib, Tucatinib, or Neratinib is generally insufficient. Since drug AEs and radiotherapy toxicities do not typically overlap entirely, concurrent use may be considered individually if there is a clear indication for radiotherapy. However, concomitant use is not recommended when the radiotherapy field involves the bowel, such as in abdominal or pelvic irradiation, and close monitoring for diarrhea is required.

**Consensus 15: Evidence regarding the efficacy and safety of combining palliative radiotherapy for extracranial lesions with Pyrotinib, Tucatinib, or Neratinib remains insufficient. This consensus suggests that if radiotherapy is indicated during systemic treatment, concomitant use with radiotherapy may be considered. However, concomitant use is not recommended for patients whose radiotherapy fields involve the intestines, particularly during abdominopelvic irradiation, and close monitoring and management of AEs such as diarrhea should be emphasized**.

#### 4.2.7 Advanced ADC: T‐DM1 and Brain Radiotherapy

T‐DM1 has demonstrated efficacy in metastatic BC and brain metastases, as shown in the EMILIA and KAMILLA studies. Key severe AEs include thrombocytopenia and elevated transaminases. With regard to cranial radiotherapy, multiple retrospective studies and meta‐analyses suggest that concurrent use of T‐DM1 with SRT or SRS may significantly increase the risk of RN, with Grade≥3 RN observed in some cohorts [48, 49, 50, 51, 52]. Studies comparing concurrent versus non‐concurrent strategies found the former associated with a higher risk of symptomatic RN, particularly in large lesions [51]. Current data do not suggest a clear survival benefit for concurrent ADC administration [53]. Evidence for extracranial radiotherapy is sparse, with only small case series suggesting no significant toxicity with hypofractionated palliative radiotherapy for bone metastases [49].

**Consensus 16: Based on evidence that the concurrent use of T‐DM1 and palliative brain radiotherapy significantly increases the risk of RN in patients with brain metastases, particularly in those with larger lesion volumes, this consensus does not recommend the combination of these two treatments. Brain metastasis volume has been identified as an independent risk factor for RN, with larger lesions conferring substantially higher risk**.

#### 4.2.8 Advanced ADC: T‐DXd and Radiotherapy

T‐DXd is used for HER2‐positive and HER2‐low metastatic BC based on the DESTINY‐Breast series, where interstitial lung disease (ILD) is a critical AE to monitor [54, 55, 56, 57]. It has shown intracranial control and survival benefits in brain metastasis populations [58]. Evidence for its combination with radiotherapy is accumulating: while the DESTINY‐Breast trials restricted ablative radiotherapy, they allowed some palliative radiotherapy without reporting significant safety signals [54, 55, 56, 57]. The prospective COMBART cohort suggested that radiotherapy during T‐DXd treatment is generally tolerable, with good disease control observed in oligoprogression combined with SRT [59]. Retrospective studies also suggest no significant increase in acute toxicity when combined with radiotherapy for bone or brain lesions [60, 61, 62].

Notably, some retrospective studies suggest that concurrent cranial radiotherapy and ADCs may increase the risk of symptomatic RN, which remained an independent factor after multivariate adjustment [51, 52, 53]. Current research shows no clear survival advantage for concurrent over non‐concurrent ADC [53]. Therefore, intracranial combination therapy should emphasize risk mitigation, while extracranial combination can be approached more proactively with caution regarding pulmonary toxicity.

**Consensus 17: Based on current evidence, concurrent use of T‐DXd and radiotherapy for intracranial lesions may increase the risk of symptomatic RN, with data suggesting that this risk is further elevated in patients with larger brain metastases, as also noted in the context of T‐DM1. This consensus recommends that for radiotherapy targeting brain metastases in metastatic BC, careful assessment is advised, and concomitant use with T‐DXd should be avoided**.

**Consensus 18: Based on existing evidence, the combination of T‐DXd and radiotherapy for extracranial oligometastatic lesions demonstrates favorable safety and certain efficacy. This consensus recommends concomitant use in such scenarios. However, evidence regarding the risk of ILD when T‐DXd is combined with thoracic radiotherapy remains insufficient; cautious evaluation is advised, and concurrent use should be avoided**.

#### 4.2.9 Advanced HR‐Positive: CDK4/6 Inhibitors With Radiotherapy

The addition of CDK4/6 inhibitors to ET has markedly improved survival in HR‐positive/HER2‐negative advanced BC, yet about half of patients require radiotherapy for disease control or symptom relief. Major Phase III trials, including MonarchE and the PALOMA series, provide limited guidance on optimal sequencing with radiotherapy. A Canadian multicenter retrospective study by Abdulla et al. showed no significant difference in ≥grade 2 non‐hematologic toxicity between concurrent and sequential approaches, although grade 3 diarrhea in concurrent treatment suggests withholding CDK4/6 inhibitors during intestinal irradiation and monitoring hematologic toxicity with pelvic fields [63]. The prospective COMBART cohort reported effective pain relief and manageable toxicity with concurrent bone radiotherapy [64]. Kubeczko et al. similarly found comparable ≥grade 3 toxicity between strategies, supporting safety and strong local control in oligometastatic disease [65]. A review of 288 patients noted higher neutropenia rates with concurrent therapy but no increase in dose reductions, concluding that routine CDK4/6 interruption during radiotherapy may be unnecessary [66].

**Consensus 19: The concurrent combination of radiotherapy and CDK4/6 inhibitors in advanced BC is generally safe with manageable toxicity. This consensus recommends considering the concurrent use of CDK4/6 inhibitors during palliative radiotherapy for metastatic lesions, particularly bone metastases. To ensure continuous control of lesions outside the radiation field, interruption of CDK4/6 inhibitor therapy during radiotherapy should be minimized whenever possible. SRT and/or hypofractionated IMRT with small target volumes are preferred. Given the varying characteristics of different metastatic sites in palliative settings, this consensus suggests stratified management based on the irradiated site: concurrent use may be considered for oligometastatic disease such as bone or brain metastases treated with stereotactic body radiation therapy or SRS and CDK4/6 inhibitors; caution is advised when combining radiotherapy with pelvic or multiple vertebral irradiation, with close attention to managing hematologic toxicity, especially during the first two weeks of CDK4/6 inhibitor therapy; for intestinal irradiation, it is recommended to temporarily suspend certain CDK4/6 inhibitors, such as Abemaciclib**.

#### 4.2.10 Advanced gBRCA‐Mutated: PARP Inhibitors

PARP inhibitors are vital for gBRCA1/2‐mutated metastatic BC, including advanced TNBC [67]. Integration with radiotherapy is currently primarily sequential, largely because evidence for the safety and benefit of concomitant administration is limited. Existing evidence comes from sequential models and early exploratory research: OlympiA confirmed the safety of Olaparib after radiotherapy [27]; the RADIOPARP Phase I study reported Grade ≥3 radiation dermatitis in 8.3% (2/24) and Grade ≥3 lymphopenia in 33.3% (8/24) with concurrent administration [28]. In the metastatic setting, evidence for concomitant palliative radiotherapy plus PARP inhibitor is insufficient to support routine use [28].

**Consensus 20: For patients with metastatic BC harboring gBRCA1/2 mutations, this consensus recommends the sequential use of PARP inhibitors and radiotherapy as standard practice. Concomitant use of PARP inhibitors with radiotherapy should be limited to small‐field, low‐dose regimens and implemented cautiously only within the framework of prospective clinical studies or after comprehensive evaluation by a multidisciplinary team. Strict monitoring and management of hematologic toxicity and radiation‐related injuries such as dermatitis and pneumonitis are essential**.

#### 4.2.11 Advanced HR‐Positive: Everolimus

Everolimus plus ET improves progression‐free survival (PFS) in specific HR‐positive/HER2‐negative advanced BC populations, as shown in the BOLERO‐2 trial. However, there are no prospective registration trials or clinical evidence for the combination of Everolimus and radiotherapy in advanced BC. Given that Everolimus can cause or exacerbate pulmonary toxicity, potential risks must be carefully considered if used concurrently with thoracic radiotherapy [68, 69].

**Consensus 21: Based on the lack of research evidence supporting the routine use of Everolimus combined with radiotherapy for synergistic effects in advanced BC, and considering the potential increased risk of pulmonary fibrosis with concomitant use, this consensus does not recommend its coadministration with thoracic radiotherapy**.

#### 4.2.12 Advanced HR‐Positive: PI3K/AKT/mTOR Pathway Inhibitors (Alpelisib, Inavolisib, Capivasertib)

Alpelisib is indicated for PIK3CA‐mutated HR‐positive/HER2‐negative advanced BC, as supported by the SOLAR‐1 and BYLieve trials [70]. While early studies in other cancers (e.g., head and neck) suggest tolerability with radiotherapy [71], there is a lack of safety and efficacy data for BC. Inavolisib is approved in China for PIK3CA‐mutated HR‐positive/HER2‐negative advanced BC, but data on radiotherapy combination are missing. Capivasertib is used for HR‐positive/HER2‐negative advanced BC with specific genetic alterations, but clear clinical evidence for radiotherapy combination is also lacking [72, 73, 74].

**Consensus 22: Given the lack of clinical evidence on the efficacy and safety of combining Alpelisib, Inavolisib, or Capivasertib with radiotherapy for advanced BC, this consensus advises that such combinations be used with caution and only in patients expected to tolerate them. Prospective studies are warranted to further investigate their use**.

#### 4.2.13 Advanced TNBC: Immunotherapy (Pembrolizumab, Toripalimab, Camrelizumab)

For advanced TNBC, KEYNOTE‐355 and TORCHLIGHT confirmed that chemotherapy plus immunotherapy improves PFS and OS in PD‐L1‐positive populations. However, whether adding radiotherapy provides a survival benefit remains without large‐scale randomized evidence. Existing data on radio‐immunotherapy in metastatic BC come from small prospective studies, suggesting that Pembrolizumab plus radiotherapy is generally tolerable, with local control benefits and occasional abscopal‐like responses [75, 76, 77, 78]. Nevertheless, optimal radiotherapy fractionation, target volume, dose, and sequencing remain unestablished. Toripalimab significantly improved PFS in PD‐L1‐positive (CPS ≥1) advanced TNBC, but direct studies on radiotherapy combination are absent [79]. Camrelizumab has shown improved pCR in TNBC [80], but systematic data for the metastatic setting and radiotherapy combination are lacking.

**Consensus 23: Although there is currently no prospective research evidence supporting the synergistic efficacy of combining ICIs with radiotherapy for the treatment of advanced BC, the established safety of combined radiotherapy and immunotherapy across multiple tumor types, along with emerging clinical benefit trends observed with Pembrolizumab plus radiotherapy in advanced BC, supports cautious use of the combination in patients likely to tolerate them. This consensus recommends conducting relevant prospective studies to further evaluate this approach**.

#### 4.2.14 Advanced TNBC/HR‐Positive: Sacituzumab Govitecan (SG)

SG has demonstrated survival benefits in advanced TNBC and HR‐positive/HER2‐negative BC in the ASCENT and TROPiCS‐02 trials, with primary toxicities being hematological and diarrhea [81, 82]. Evidence for SG with radiotherapy is limited to small retrospective studies, most of which suggest acceptable safety and efficacy signals in brain or bone metastasis [83, 84, 85, 86]. Some cohorts reported high ORR and potential survival improvement with the combination [85, 86], with no significant difference in moderate‐to‐severe acute toxicity between concurrent and sequential strategies [84]. Notably, while concurrent cranial radiotherapy and ADCs may increase RN risk, the SG‐related subgroup analysis was not statistically significant, requiring further clarification [51].

**Consensus 24: Available retrospective studies suggest that combining SG with radiotherapy does not lead to a significant increase in toxicity for patients with advanced BC, and may offer potential survival benefits specifically in those with brain metastases. Accordingly, this consensus recommends further clinical studies to clarify the synergistic efficacy and safety of this combination. For radiotherapy targeting brain lesions, enhanced monitoring for the risk of RN is advised**.

#### 4.2.15 Advanced TNBC/HR‐Positive: Novel TROP‐2 ADCs (SKB‐264, Dato‐DXd)

Novel TROP‐2 ADCs such as SKB‐264 and Dato‐DXd are being used in later‐line therapy for advanced TNBC or HR‐positive/HER2‐negative BC, but systematic clinical data on radiotherapy combination are missing. Given their shared toxicity profiles with SG, clinical combination should prioritize research settings.

**Consensus 25: Based on the tolerable safety profile observed with the combination of SG and radiotherapy in small cohort studies, and considering the similarities between SG and novel TROP‐2‐directed ADCs, this consensus suggests careful consideration of such combination regimens in clinical practice and recommends conducting additional prospective studies to clarify their efficacy and safety**.

#### 4.2.16 Advanced BC: Bevacizumab

The strongest evidence for Bevacizumab comes from brain metastases: the BEEP trial showed a trend toward improved brain‐specific PFS with WBRT plus induction Bevacizumab versus WBRT alone [87]; the Phase I REBECA study also found Bevacizumab plus WBRT to be well tolerated [88]. Data from other cancers suggest that combining Bevacizumab with cranial radiotherapy is feasible and may reduce radiation necrosis or cerebral edema [89, 90, 91]. However, in extracranial radiotherapy, particularly for abdominal or pulmonary lesions, data from other tumors suggest an increased risk of gastrointestinal injury or perforation, as well as RP [92, 93]. In the adjuvant BC setting, prospective studies suggest acceptable short‐ and long‐term toxicity with concurrent use [94, 95, 96, 97].

**Consensus 26: Based on its favorable safety and tolerability profile, this consensus recommends that Bevacizumab be combined concurrently with WBRT or SRT in BC patients with brain metastases. Cautious evaluation is advised when Bevacizumab is combined with radiotherapy for extracranial lesions, particularly those involving the lung or gastrointestinal tract**.

#### 4.2.17 Advanced BC: Apatinib

Apatinib has limited direct evidence in BC, consisting mostly of case reports or small retrospective series. Some cases suggest that low‐dose Apatinib plus hypofractionated radiotherapy can achieve local response [98]. Evidence from other tumors suggests concurrent use with WBRT or SRT might improve intracranial control and survival, but the level of evidence is low and requires cautious extrapolation [99, 100]. Overall, high‐quality evidence for routine recommendation is lacking, with particular concern for patients at risk of bleeding or mucosal injury.

**Consensus 27: Although direct evidence in BC is limited, based on safety across tumor type and potential efficacy, it is suggested that the combination of Apatinib with radiotherapy may be considered for BC patients. Caution is advised, especially for lesions at risk of bleeding (e.g., superficial ulcers). Prospective studies are recommended to further validate this approach**.

#### 4.2.18 Advanced HR‐Positive: Oral SERDs (Elacestrant)

Oral selective estrogen receptor degraders (SERDs) like elacestrant are used for HR‐positive/HER2‐negative metastatic BC with ESR1 mutations. Currently, there is no public clinical evidence or systematic data regarding their combination with radiotherapy. In practice, risk management should be based on toxicity profiles and the site of irradiation.

**Consensus 28: Given that the toxicity profiles of radiotherapy and SERD do not overlap, concomitant use may be considered if radiotherapy is indicated during systemic therapy, such as for curative treatment of oligometastases or palliative treatment of bone metastases. Prospective studies are recommended**.

## 5 Discussion

This multidisciplinary expert consensus provides a comprehensive framework for the clinical integration of radiotherapy and novel systemic agents in BC. In the era of precision medicine, the role of radiotherapy has expanded from a local control modality to an essential component of integrated therapeutic strategies. By combining radiotherapy with ET, HER2‐targeted agents, CDK4/6 inhibitors, ADCs, immunotherapy, and PARP inhibitors, the current treatment landscape is transitioning from traditional sequential models toward synergistic combinations. This evolution aligns with the rapid clinical development of systemic therapies and the increasing need for optimized multimodal management.

Clinically, the synergy between radiotherapy and various systemic agents is highly heterogeneous, requiring individualized strategies rather than a one‐size‐fits‐all approach. For instance, while CDK4/6 inhibitors and PARP inhibitors enhance the sensitivity of tumor cells to radiation, their clinical application must be carefully timed to balance efficacy and toxicity. Similarly, the integration of immunotherapy and radiotherapy relies on the potential of radiation to prime the immune response, yet the optimal clinical window for this combination remains under investigation. These observations emphasize that combined treatment plans should be designed based on the specific safety profiles and clinical indications of each drug class.

The clinical value of these combinations varies across different disease stages. In early‐stage BC, the primary goal is to reduce locoregional recurrence while maintaining systemic treatment intensity. This consensus notes that while ET is routinely administered concurrently with radiotherapy, the concurrent use of CDK4/6 inhibitors is still largely restricted to clinical trials or specific cases, reflecting a gap between real‐world practice and high‐level randomized evidence. For HER2‐positive patients, although concurrent treatment with Trastuzumab and radiotherapy is well‐tolerated, the use of ADCs such as T‐DM1 requires vigilant monitoring for skin and pulmonary toxicities. These findings highlight that as treatment intensity increases in the adjuvant setting, managing cumulative toxicity becomes a priority for clinicians. For TNBC, the clinical focus is on maximizing the response in patients with residual disease. While combining Capecitabine or immunotherapy with radiotherapy shows potential survival benefits, most available data are derived from Phase II or retrospective studies. The optimal sequencing of immunotherapy and radiotherapy is particularly debated, as clinical evidence is not yet sufficient to define a standard of care for postoperative integration. This remains a critical area for future prospective research.

In the metastatic setting, the priority is to integrate local radiotherapy without interrupting effective systemic therapy. This consensus supports the use of radiotherapy for oligometastatic disease to improve PFS. In patients with brain metastases, the combination of HER2‐targeted TKIs and radiotherapy is supported by relatively mature evidence. However, caution is advised when using ADCs concurrently with brain radiotherapy due to an observed increase in the risk of radiation necrosis. This underscores the necessity for organ‐specific safety assessments when choosing systemic agents for patients undergoing radiation.

Safety management is the cornerstone of successful combined therapy. The consensus emphasizes that the clinical bottleneck has shifted from achieving efficacy to balancing treatment‐related toxicities, such as cardiotoxicity, pneumonitis, and radionecrosis. The use of advanced radiotherapy techniques, including IMRT and hypofractionation, is essential to minimize doses to normal tissues, thereby providing a technical safeguard for concurrent systemic administration. Future clinical trials should consistently include dosimetric parameters as key endpoints to better define safety margins.

Furthermore, the “Multi‐dimensional Decision Model” proposed in this consensus provides a practical tool for multidisciplinary teams (MDTs). By integrating disease stage, molecular subtype, and patient‐specific factors, this model encourages a shift from protocol‐driven to strategy‐driven decision‐making.

Despite the progress outlined above, several clinical challenges persist. Most current strategies have not been validated in Phase III randomized controlled trials, and available real‐world evidence remains heterogeneous. Future research should prioritize prospective studies to confirm the efficacy of key combination approaches, the identification of reliable clinical biomarkers to better select patients most likely to benefit, optimization of radiotherapy dose and fractionation schedules in the setting of immunotherapy and ADCs, and the integration of digital technologies and artificial intelligence to improve toxicity monitoring and prediction.

In conclusion, the integration of radiotherapy and novel systemic agents represents a significant advancement in BC care. Its value lies not only in improving local control but also in the potential for systemic synergy and treatment de‐escalation. Through continued clinical research and precise patient stratification, the medical community can refine these integrated paradigms to move toward truly individualized BC therapy.

## Funding

The consensus was supported by Noncommunicable Chronic Diseases‐National Science and Technology Major Project (2023ZD0502300 [2023ZD0502301, 2023ZD0502302, 2023ZD0502303]), Sichuan Science and Technology Program (2025ZDZX0012), and Sichuan Provincial Engineering Research Center of Intelligent Diagnosis and Treatment of Breast Diseases.

## Conflicts of Interest

The authors declare no conflicts of interest.

## Consensus Panelist Group

**Steering Committee**: Jing Jing (West China Hospital, Sichuan University); Xiaobo Huang (Sun Yat‐sen Memorial Hospital, Sun Yat‐sen University); Shusen Wang (Sun Yat‐sen University Cancer Center); Lei Liu (West China Hospital, Sichuan University).

**Chief Advisory Expert Committee**: Jinming Yu (Shandong Cancer Hospital and Institute, Shandong First Medical University and Shandong Academy of Medical Sciences); Binghe Xu (National Cancer Center/National Clinical Research Center for Cancer/Cancer Hospital, Chinese Academy of Medical Sciences and Peking Union Medical College); Zefei Jiang (Fifth Medical Center of Chinese PLA General Hospital); Jin Zhang (Tianjin Medical University Cancer Institute and Hospital); Yongmei Yin (The First Affiliated Hospital of Nanjing Medical University).

**Guiding Expert Committee**: Shulian Wang (National Cancer Center/National Clinical Research Center for Cancer/Cancer Hospital, Chinese Academy of Medical Sciences and Peking Union Medical College); Jiayi Chen (Ruijin Hospital, Shanghai Jiao Tong University School of Medicine); Cuizhi Geng (Fourth Hospital of Hebei Medical University); Qiang Liu (Sun Yat‐sen Memorial Hospital, Sun Yat‐sen University); Guojun Zhang (The Third Affiliated Hospital of Kunming Medical University).

**Writing Expert Committee**: Jing Jing (West China Hospital, Sichuan University); Xiaobo Huang (Sun Yat‐sen Memorial Hospital, Sun Yat‐sen University); Shusen Wang (Sun Yat‐sen University Cancer Center); Xin Wu (West China Hospital, Sichuan University); Lei Liu (West China Hospital, Sichuan University); Yanxia Zhao (Union Hospital, Tongji Medical College, Huazhong University of Science and Technology); Jianli Zhao (Sun Yat‐sen Memorial Hospital of Sun Yat‐sen University); Jun Zhang (The Fourth Hospital of Hebei Medical University); Xiaoli Yu (Fudan University Shanghai Cancer Center); Huihua Xiong (Tongji Hospital, Tongji Medical College, Huazhong University of Science and Technology); Wen Xia (Sun Yat‐sen University Cancer Center), Yanchu Li (West China Hospital, Sichuan University), Chunling Jiang (Shanghai General Hospital, Shanghai Jiao Tong University School of Medicine); Zhenyu He (Sun Yat‐sen University Cancer Center); Wenyan Chen (Nanchang People's Hospital); Lu Cao (Ruijin Hospital, Shanghai Jiao Tong University School of Medicine); Qiufan Zheng (Sun Yat‐sen University Cancer Center); Wenyi Zhou (Sun Yat‐sen Memorial Hospital, Sun Yat‐sen University); Jie Lan (West China Hospital, Sichuan University).

**Expert Panel**: Zhuofei Bi (Sun Yat‐sen Memorial Hospital, Sun Yat‐sen University); Lu Cao (Ruijin Hospital, Shanghai Jiao Tong University School of Medicine); Jie Chen (West China Hospital, Sichuan University); Jiayi Chen (Ruijin Hospital, Shanghai Jiao Tong University School of Medicine); Ru Chen (Hainan General Hospital); Wenyan Chen (Nanchang People's Hospital); Zhenggui Du (West China Hospital, Sichuan University); Fang Dong (Gansu Provincial Cancer Hospital); Lu Gan (The First Affiliated Hospital of Chongqing Medical University); Cuizhi Geng (Fourth Hospital of Hebei Medical University); Chunfang Hao (Tianjin Cancer Hospital Airport Hospital); Xiaopeng Hao (Chinese PLA General Hospital); Zhenyu He (Sun Yat‐sen University Cancer Center); Zhiyao He (West China Hospital, Sichuan University); Xiaobo Hu (Hunan Cancer Hospital); Hong Hu (Shenzhen People's Hospital); Ying Hu (Hunan Cancer Hospital); Xiaobo Huang (Yat‐sen Breast Tumor Hospital, Sun Yat‐sen Memorial Hospital, Sun Yat‐sen University); Suning Huang (Guangxi Medical University Cancer Hospital); Wei Huang (Shandong Cancer Hospital and Institute, Shandong First Medical University); Chunling Jiang (Shanghai General Hospital, Shanghai Jiao Tong University School of Medicine); Haiman Jing (The University of Hong Kong‐Shenzhen Hospital); Jing Jing (West China Hospital, Sichuan University); Jie Lan (West China Hospital, Sichuan University); Huihui Li (Shandong Cancer Hospital and Institute, Shandong First Medical University); Junjie Li (Fudan University Shanghai Cancer Center); Man Li (The Second Hospital of Dalian Medical University); Yanchu Li (West China Hospital, Sichuan University); Yuntao Li (The Fourth Hospital of Hebei Medical University); Lei Liu (West China Hospital, Sichuan University); Shu Liu (Affiliated Hospital of Guizhou Medical University); Tao Liu (Sun Yat‐sen University Cancer Center); Xinlan Liu (People's Hospital of Ningxia Hui Autonomous Region); Zhikun Liu (The Fourth Hospital of Hebei Medical University); Ting Luo (West China Hospital, Sichuan University); Yongkui Lu (Guangxi Medical University Cancer Hospital); Yufei Lu (Henan Cancer Hospital); Jie Ma (Tangshan City People's Hospital); Quchang Ouyang (Hunan Cancer Hospital); Peijian Peng (The Fifth Affiliated Hospital of Sun Yat‐sen University); Bin Shao (Peking University Cancer Hospital); Yuhua Song (The Affiliated Hospital of Qingdao University); Bing Sun (The Fifth Medical Center of Chinese PLA General Hospital); Tiantian Zhai (Cancer Hospital of Shantou University Medical College); Yuee Teng (The First Hospital of China Medical University); Qin Tong (The First Affiliated Hospital of Nanhua University); Ruozheng Wang (Affiliated Tumor Hospital of Xinjiang Medical University); Shulian Wang (National Cancer Center/National Clinical Research Center for Cancer/Cancer Hospital, Chinese Academy of Medical Sciences and Peking Union Medical College); Ting Wang (Xijing Hospital of Air Force Medical University); Xiaohong Wang (Tangshan City People's Hospital); Yu Wang (Shanxi Cancer Hospital); Xin Wu (West China Hospital, Sichuan University); Junxin Wu (Fujian Cancer Hospital); Junyan Wu (Sun Yat‐sen Memorial Hospital, Sun Yat‐sen University); Wen Xia (Sun Yat‐sen University Cancer Center); Yaoxiong Xia (Yunnan Cancer Hospital); Xiaohua Zeng (Chongqing University Cancer Hospital); Huihua Xiong (Tongji Hospital, Tongji Medical College, Huazhong University of Science and Technology); Hongwei Yang (Suining Central Hospital); Huawei Yang (Guangxi Medical University Cancer Hospital); Jin Yang (First Affiliated Hospital of Xi'an Jiao Tong University); Weifang Yang (Taizhou Hospital of Zhejiang Province); Jing Yao (Union Hospital, Tongji Medical College, Huazhong University of Science and Technology); Guolin Ye (The First People's Hospital of Foshan); Haijun Yu (Zhongnan Hospital of Wuhan University); Xiaoli Yu (Fudan University Shanghai Cancer Center); Anqin Zhang (Guangdong Women and Children's Hospital); Guojun Zhang (The Third Affiliated Hospital of Kunming Medical University); Jun Zhang (The Fourth Hospital of Hebei Medical University); Na Zhang (Liaoning Cancer Hospital); Qiang Zhang (Liaoning Cancer Hospital); Yuanqi Zhang (Affiliated Hospital of Guangdong Medical University); Bing Zhao (Affiliated Tumor Hospital of Xinjiang Medical University); Jianli Zhao (Sun Yat‐sen Memorial Hospital of Sun Yat‐sen University); Lina Zhao (Xijing Hospital of Air Force Medical University); Yanxia Zhao (Union Hospital, Tongji Medical College, Huazhong University of Science and Technology); Qiufan Zheng (Sun Yat‐sen University Cancer Center); Ning Zou (Hubei Cancer Hospital).
